# Sparsity-Aware Noise Subspace Fitting for DOA Estimation

**DOI:** 10.3390/s20010081

**Published:** 2019-12-21

**Authors:** Chundi Zheng, Huihui Chen, Aiguo Wang

**Affiliations:** School of Electronic Information Engineering, Foshan University, Foshan 528231, Guangdong, China; cdzheng@fosu.edu.cn (C.Z.); hhchen@fosu.edu.cn (H.C.)

**Keywords:** direction-of-arrival (DOA) estimation, sparse recovery, subspace fitting, array signal processing, linearly constrained quadratic programming (LCQP)

## Abstract

We propose a sparsity-aware noise subspace fitting (SANSF) algorithm for direction-of-arrival (DOA) estimation using an array of sensors. The proposed SANSF algorithm is developed from the optimally weighted noise subspace fitting criterion. Our formulation leads to a convex linearly constrained quadratic programming (LCQP) problem that enjoys global convergence without the need of accurate initialization and can be easily solved by existing LCQP solvers. Combining the weighted quadratic objective function, the $\ell_{1}$ norm, and the non-negative constraints, the proposed SANSF algorithm can enhance the sparsity of the solution. Numerical results based on simulations, using real measured ultrasonic, and radar data, show that, compared to existing sparsity-aware methods, the proposed SANSF can provide enhanced resolution with a lower computational burden.

## 1. Introduction

Direction-of-arrival (DOA) estimation is one of the key problems in array processing [1,2]. Over the last 50 years, a variety of techniques have been developed to address this problem [2,3,4,5,6,7,8,9,10,11,12,13,14], in which high resolution, low complexity, and good statistical performance are desired. Especially, the maximum likelihood (ML) method was investigated because it can achieve the Cramér-Rao low bound (CRLB) for large samples [5,15,16,17,18,19]. Although the ML method has asymptotic statistical efficiency, its computational cost is extremely high and its global solution is not guaranteed [20,21].

The noise subspace fitting (NSF) methods and their variants are popular approximations of the ML method. One of the well-known NSF methods is multiple signal classification (MUSIC), which is computationally simple and asymptotically equivalent to the ML method for uncorrelated signals [6]. In the case of correlated sources, however, MUSIC is unsatisfactory from a statistical viewpoint [5]. To deal with correlated sources, an improved NSF strategy called the optimally weighted noise subspace fitting (OWNSF) was developed to achieve the asymptotic property of ML, and the corresponding algorithm is named method of direction estimation (MODE) [5,16,21]. For uniform linear arrays (ULA), MODE can be implemented by eigen-analysis with the computational cost of $O(\frac{1}{2}\left(M+1\right)MT)$ flops, where *M* and *T* denote the number of sensors and snapshots, respectively [22]. For general array geometries, however, the implementation of MODE requires iterative non-linear search procedures (e.g., the Gauss–Newton search technique), in which an accurate initialization is crucial to avoid local minima [5,16]. An accurate initialization may be difficult when there exist closely spaced sources [16]. Therefore, MODE suffers from initialization difficulties and its global solution may not be guaranteed for general array geometries.

Based on the sparsity of the spatial spectrum, some sparsity-aware methods have been proposed recently to avoid the requirement on accurate initializations [8,9,10,23,24,25,26]. Among them, a representative method is the global matched filter (GMF), which is robust according to correlated and even coherent sources [27,28]. GMF is a quadratic minimization with $\ell_{1}$ norm regularization, so it has an unique global minimum [27]. In addition, by weighting the $\ell_{1}$ norm penalty item, a weighted GMF (WGMF) was proposed in [11]. However, WGMF requires solving a second order cone programming (SOCP), for which the computational cost of each iteration is on the order of $O(K^{3}P^{3})$ complex operations with the interior-point algorithm [29], where *P* and *K* are the length of the sparse signal and the number of sources, respectively. A hyperparameter-free estimator, called the likelihood-based estimation of sparse parameters (LIKES), was also developed from the ML criterion [30]. The computational cost per iteration of LIKES is on the order of O(2PMT) complex operations (typically M≪P for sparse representation) [31]. From a computational point of view, LIKES is attractive when MT is moderate. However, LIKES also suffers from a large computational cost problem when MT is large. A gridless sparse method called gridless sparse iterative covariance-based estimation (GL-SPICE, GLS) can estimate DOA without grid selection, which is very practical to relieve the grid mismatch problem of spare recovery [32,33]. The literature [34] addressed the DOA estimation with a block sparse reconstruction method in the presence of unknown mutual coupling. But GLS [33] and the method presented in [34] can only deal with linear arrays.

In this paper, we propose a sparsity-aware noise subspace fitting (SANSF) algorithm by extending the OWNSF criterion [16] to achieve high-resolution DOA estimation for general array geometries. The extension of the OWNSF criterion under the sparse representation framework leads to a new quadratic objective function that contains a positive semidefinite Hessian matrix. The total power and the non-negative property of the spectral components are used as the constraints of the objective function. In this way, the proposed SANSF algorithm is converted into a convex linearly constrained quadratic programming (LCQP) problem without the requirement of an accurate initialization. Furthermore, the proposed SANSF algorithm can be efficiently solved by many existing LCQP solvers [35,36,37]. Compared to SOCP, LCQP is computationally more efficient [38]. Simulations and experimental results using real measured ultrasonic data and radar data demonstrate that, compared to the existing sparsity-aware methods, SANSF can achieve higher resolution with relatively lower computational cost.

The proposed SANSF algorithm has three attractive features: (1) Its convex quadratic objective function enjoys global convergence without an accurate initialization requirement; (2) the SANSF problem is an LCQP so that it can be easily solved by efficient solvers with low computational costs; (3) not only the weighted quadratic objective function but also the $\ell_{1}$ norm and the non-negative constraints play the role of encouraging sparseness, which would be very helpful to improve the resolution.

The remainder of this paper is organized as follows. In Section 2, the signal model is introduced. In Section 3, the proposed SANSF algorithm is formulated, and its advantages are analyzed. In Section 4, experimental results on simulated and real measured data are presented to illustrate the performance of the proposed algorithm. Concluding remarks are given in Section 5.

## 2. Signal Model

### 2.1. Array Processing Model

Assume that there are *M* sensors with general array geometries and *K* far-field uncorrelated narrowband signals $\left\{s_{k}\left(t\right),k=1,2,\cdots ,K\right\}$, where the signals are impinging on the array from distinct directions $\left\{\theta_{k},k=1,2,\cdots ,K\right\}$. The model of the received signal can be expressed as:

(1) y(t)=As(t)+n(t),t=1,2,⋯,T,

where y(t), s(t), n(t), and *T* denote the measurement vector, the signal vector, the noise vector, and the number of snapshots, respectively. The matrix $A\in C^{M\times K}$ is the array response matrix given by $A=[a\left(\theta_{1}\right),\cdots ,a\left(\theta_{K}\right)]$, where $a\left(\theta_{k}\right)\in C^{M\times 1}$ is the so-called steering vector depending on the array geometry. n(t) is an additive noise vector with zero-mean and covariance matrix $\sigma^{2}I$. The noise n(t) is assumed to be uncorrelated to s(t).

The covariance matrix of y(t) can be expressed as

(2) $$\begin{array}{ccc} R & = & E\{y\left(t\right)y^{H}\left(t\right)\}\\ & = & AE\left\{s\left(t\right)s^{H}\left(t\right)\right\}A^{H}+\sigma^{2}I_{M}\\ & = & {\mathrm{AR}}_{S}A^{H}+\sigma^{2}I_{M}, \end{array}$$

where $R_{S}=E\left\{s\left(t\right)s^{H}\left(t\right)\right\}$, $I_{M}$ is a M×M identity matrix, and ${\left(\cdot \right)}^{H}$ denotes the conjugate transpose.

Under the assumption of uncorrelated signals, the eigen-decomposition of R can be written as

(3) $$\begin{array}{c} R=\sum_{m=1}^{M}\lambda_{m}u_{m}u_{m}^{H}=U_{S}\Lambda_{S}U_{S}^{H}+\sigma^{2}U_{N}U_{N}^{H}, \end{array}$$

where $\lambda_{1}\geq \lambda_{2}\geq \cdots \geq \lambda_{K}\geq \lambda_{K+1}=\cdots =\lambda_{M}=\sigma^{2}$ are the eigenvalues of R, $\Lambda_{S}=\mathrm{diag}\left\{\lambda_{1},\cdots ,\lambda_{K}\right\}$, $\Lambda_{N}=\mathrm{diag}\left\{\lambda_{K+1},\cdots ,\lambda_{M}\right\}$, $U_{S}$ and $U_{N}$ span the signal and noise subspaces, respectively, diag{·} denotes the diagonal matrix. Consistent estimates of the signal and noise subspaces can be obtained by the eigendecomposition of the sample covariance matrix

(4) $$\begin{array}{ccc} \hat{R} & = & \frac{1}{T}\sum_{t=1}^{T}y\left(t\right)y^{H}\left(t\right)\\ & = & \sum_{m=1}^{M}{\hat{\lambda }}_{m}{\hat{u}}_{m}{\hat{u}}_{m}^{H}\\ & = & {\hat{U}}_{S}{\hat{\Lambda }}_{S}{\hat{U}}_{S}^{H}+{\hat{U}}_{N}{\hat{\Lambda }}_{N}{\hat{U}}_{N}^{H}, \end{array}$$

where ${\hat{U}}_{S}=\left[{\hat{u}}_{1},\cdots ,{\hat{u}}_{K}\right]$, ${\hat{U}}_{N}=\left[{\hat{u}}_{K+1},\cdots ,{\hat{u}}_{M}\right]$, ${\hat{\Lambda }}_{S}=\mathrm{diag}\left\{{\hat{\lambda }}_{1},\cdots ,{\hat{\lambda }}_{K}\right\}$, ${\hat{\Lambda }}_{N}=\mathrm{diag}\left\{{\hat{\lambda }}_{K+1},\cdots ,{\hat{\lambda }}_{M}\right\}$, and ${\hat{\lambda }}_{m}$ and ${\hat{u}}_{m}$ are the estimates of $\lambda_{m}$ and $u_{m}$, respectively.

### 2.2. OWNSF and Its Sparse Representation

Next, the OWNSF criterion and its sparse representation are reviewed. The OWNSF criterion can be expressed by minimizing the following function [5,16]:

(5) $$\begin{array}{c} f=∥{\hat{U}}_{N}^{H}AW_{\mathrm{opt}}^{1/2}{∥}_{F}^{2} \end{array}$$

where $W_{\mathrm{opt}}=A^{†}U_{S}{\left(\Lambda_{S}-\sigma^{2}I_{M}\right)}^{2}\Lambda_{S}^{-1}U_{S}^{H}A^{†H}$, ${∥\cdot ∥}_{F}$, and ${\left(\cdot \right)}^{†}$ denote the Frobenius norm and the pseudo-inverse, respectively. In practice, for general array geometries, an iterative non-linear search procedure [5,16] can be used to implement OWNSF after replacing $W_{\mathrm{opt}}$ with its estimate ${\hat{W}}_{\mathrm{opt}}=A^{†}\left(\theta_{0}\right){\hat{U}}_{S}{\left({\hat{\Lambda }}_{S}-{\hat{\sigma }}^{2}I_{M}\right)}^{2}{\hat{\Lambda }}_{S}^{-1}{\hat{U}}_{S}^{H}A^{†H}\left(\theta_{0}\right)$, where $\theta_{0}$ is the initial estimate of the DOAs.

The following relationship was verified in [5,16]:

(6) $$A^{†}U_{S}{\left(\Lambda_{S}-\sigma^{2}I_{M}\right)}^{2}\Lambda_{S}^{-1}U_{S}^{H}A^{†H}=R_{S}A^{H}R^{-1}AR_{S}.$$

Substituting Equation (6) into Equation (5), we have

(7) $$\begin{array}{ccc} f & = & ∥{\hat{U}}_{N}^{H}AW_{\mathrm{opt}}^{1/2}{∥}_{F}^{2}\\ & = & \mathrm{Tr}\{W_{\mathrm{opt}}A^{H}{\hat{U}}_{N}{\hat{U}}_{N}^{H}A\}\\ & = & \mathrm{Tr}\{R_{S}A^{H}R^{-1}AR_{s}A^{H}{\hat{U}}_{N}{\hat{U}}_{N}^{H}A\}\\ & = & \mathrm{Tr}\{R^{-1}AR_{S}A^{H}{\hat{U}}_{N}{\hat{U}}_{N}^{H}AR_{S}A^{H}\}, \end{array}$$

where Tr{·} denotes the trace of a matrix.

Under the assumption of the uncorrelated signals (The proposed SANSF algorithm is robustness to this assumption. As can be seen from the experimental section, SNASF works well even if there are some correlated sources.), a sparse representation of $AR_{S}A^{H}$ can be expressed as

(8) $$\begin{array}{ccc} AR_{S}A^{H} & = & B\Gamma B^{H}, \end{array}$$

where

(9) $$\begin{array}{ccc} \Gamma & ≜ & \left[\begin{array}{cccc} \gamma_{1} & 0 & \cdots & \cdots \\ 0 & \gamma_{2} & 0 & \cdots \\ \vdots & 0 & \ddots & \vdots \\ 0 & \cdots & \cdots & \gamma_{P} \end{array}\right]\in R^{P\times P}, \end{array}$$

(10) $$\gamma_{p}≜E\left\{s_{p}\left(t\right)s_{p}^{*}\left(t\right)\right\},$$

and

(11) $$B≜\left[b_{1},\cdots ,b_{p},\cdots ,b_{P}\right]\in C^{M\times P},$$

P≫M, ${\left(\cdot \right)}^{*}$ denotes the complex conjugate, $b_{p}\in C^{M\times 1}$ is the steering vector for the *p*-th angular grid point, p=1,⋯,P, and *P* is the number of angular grid points.

The definition of $\gamma_{p}$ in Equation (10) implies that $\gamma_{p}\geq 0$. $\gamma_{p}>0$ denotes that there exists a source at the *p*-th angular grid point with the power value $\gamma_{p}$, and $\gamma_{p}=0$ if and only there is no source at the *p*-th angular grid point. Hence, the spatial spectrum can be expressed by γ, where $\gamma ={\left[\gamma_{1},\cdots ,\gamma_{P}\right]}^{T}$, with ${\left(\cdot \right)}^{T}$ denoting the transpose. The support of γ can be defined as $\Omega ≜\left\{p|\gamma_{p}>0\right\}\subseteq \left\{1,\cdots ,P\right\}$, which results in $A=B_{\Omega }$, where $B_{\Omega }$ denotes a matrix whose columns are indexed by the set Ω.

Substituting Equation (8) into Equation (7), we have

(12) $$\begin{array}{ccc} f & = & \mathrm{Tr}\{R^{-1}B\Gamma B^{H}{\hat{U}}_{N}{\hat{U}}_{N}^{H}B\Gamma B^{H}\}\\ & = & \mathrm{Tr}\{\Gamma B^{H}R^{-1}B\Gamma B^{H}{\hat{U}}_{N}{\hat{U}}_{N}^{H}B\}. \end{array}$$

According to the above derivation, it is clear that minimizing Equation (12) can be regarded as an extension of the OWNSF criterion under the sparse framework. The main difference between Equation (7) and Equation (12) is that $AR_{S}A^{H}$ (i.e., $B_{\Omega }R_{S}B_{\Omega }^{H}$) in Equation (7) is replaced with its sparse representation $B\Gamma B^{H}$ in Equation (12). Minimizing the OWNSF criterion in Equation (5) requires an initial estimate of Ω (i.e., $\theta_{0}$) and the corresponding algorithm is called MODE [5,16].

## 3. The Proposed SANSF Algorithm for DOA Estimation

In this section, we formulate the proposed SANSF algorithm and discuss its performance.

### 3.1. Sparsity-Aware Noise Subspace Fitting

By replacing R with $\hat{R}$ in Equation (12) and then minimizing Equation (12) with respecting to the unknown spatial spectrum γ, we propose the following quadratic objective function

(13) $$\begin{array}{ccc} \hat{\gamma } & = & \underset{\gamma }{\mathrm{argmin}}\mathrm{Tr}\left\{\Gamma B^{H}{\hat{R}}^{-1}B\Gamma B^{H}{\hat{U}}_{N}{\hat{U}}_{N}^{H}B\right\}\\ & = & \underset{\gamma }{\mathrm{argmin}}\mathrm{Tr}\left\{\Gamma C\Gamma D\right\}\\ & = & \underset{\gamma }{\mathrm{argmin}}\gamma^{T}G\gamma , \end{array}$$

where the last equality is derived from the diagonal characteristics of Γ, $C=B^{H}{\hat{R}}^{-1}B$, $D=B^{H}{\hat{U}}_{N}{\hat{U}}_{N}^{H}B$, $G=\mathrm{Real}(C⊙D^{T})$, ⊙ and Real(·) denote the Hadamard product and taking the real part operators, respectively. It is clear that G can be computed from the known array geometry and the measurement vector, so Equation (13) is a quadratic function of the spatial spectrum γ.

From Equations (2), (3), and (8), we have

(14) $$\mathrm{Tr}\left\{B\Gamma B^{H}\right\}=\mathrm{Tr}\left\{U_{S}\left(\Lambda_{S}-\sigma^{2}I\right)U_{S}^{H}\right\}.$$

Due to the diagonal characteristics of Γ, Equation (14) can be rewritten as

(15) $$M\sum_{p=1}^{P}\gamma_{p}=\mathrm{Tr}\left\{\Lambda_{S}-\sigma^{2}I\right\}.$$

Namely, a constraint on γ can be expressed by

(16) $${∥\gamma ∥}_{1}=1^{T}\gamma =\mathrm{Tr}\left\{\Lambda_{S}-\sigma^{2}I\right\}/M,$$

where ${∥\cdot ∥}_{1}$ denotes the $\ell_{1}$ norm and 1 is an all-one vector. The constraint in Equation (16) suggests the magnitude of the retrieved spectrum.

By combining Equation (13), Equation (16), and the non-negative property of the spectrum components, the proposed SANSF algorithm can be expressed as the following quadratic minimization with the linear constraints:

(17) $$\begin{array}{c} \min_{\gamma \geq 0}\gamma^{T}G\gamma \;s.t.\;\;1^{T}\gamma =\mathrm{Tr}\left\{\Lambda_{S}-\sigma^{2}I\right\}/M, \end{array}$$

where γ≥0 means that $\left\{\gamma_{p}\geq 0,p=1,\cdots ,P\right\}$. To solve Equation (17) in practice, $\Lambda_{S}$ and $\sigma^{2}$ need to be replaced with correspondingly consistent estimates ${\hat{\Lambda }}_{S}$ and ${\hat{\sigma }}^{2}$, respectively, where ${\hat{\sigma }}^{2}=\sum_{\hat{K}+1}^{M}{\hat{\lambda }}_{m}/\left(M-\hat{K}\right)$, and $\hat{K}$ is an estimate of *K* that can be obtained by the minimum description length (MDL) criterion [39].

The optimization problem in Equation (17) is an LCQP problem that can be solved by off-the-shelf toolbox [35,36,40]. Here, we employ the MATLAB’s *quadprog* solver to implement SANSF, in which the computational complexity is on the order of $O(8P^{3})$ real operations [41].

### 3.2. About the Initialization

The major drawback of MODE for general array geometries is the requirement for an accurate initialization to guarantee the convergence to the true solution [5,16,21,42]. Although the initialization can typically be performed by MUSIC or alternating projection (AP) [20], they may not work well when there are closely spaced sources [16]. The inaccurate initialization will seriously degrade the performance of MODE [5,16]. In contrast, SANSF proposed in Equation (17) has global convergence without any requirement for accurate initialization. We explain it in detail below.

The convexity of Equation (17) depends on the convexity of Equation (13), since the constraint in Equation (17) is linear. The sufficient condition of the convexity of Equation (13) is that the Hessian matrix $G=\mathrm{Real}(C⊙D^{T})$ is positive semidefinite. We first analyze the property of C:

(18) $$\begin{array}{ccc} C & = & B^{H}{\hat{R}}^{-1}B\\ & = & B^{H}\hat{U}{\hat{\Lambda }}^{-1/2}{\hat{\Lambda }}^{-1/2}{\hat{U}}^{H}B\\ & = & E^{H}E, \end{array}$$

where $\hat{U}=\left[{\hat{U}}_{S},{\hat{U}}_{N}\right]$, $\hat{\Lambda }=\mathrm{diag}\left\{{\hat{\lambda }}_{1},\cdots ,{\hat{\lambda }}_{M}\right\}$, and $E={\hat{\Lambda }}^{-1/2}{\hat{U}}^{H}B$. Because both $\hat{\Lambda }\in R^{M\times M}$ and $\hat{U}\in C^{M\times M}$ are full rank matrices, we have

(19) $$\begin{array}{c} \mathrm{rank}(E)=\mathrm{rank}(B)=M, \end{array}$$

where rank(·) denotes the rank of the matrix.

According to Equation (19), we can conclude that ${\mathrm{EE}}^{H}$ is nonsingular and its eigenvalues are positive numbers, which means that $E^{H}E$ has *M* positive eigenvalues and P−M zero-value eigenvalues [43]. As a result, C is positive semidefinite. Similarly, D is also positive semidefinite. Theorem 5.2.1 in [44] indicates that, if C and D are positive semidefinite, $G=\mathrm{Real}(C⊙D^{T})$ is also positive semidefinite. Based on these observations, we can conclude that the objective function of Equation (17) is convex. This means that SANSF ensures global convergence without any requirement on an accurate initialization.

### 3.3. The Effect of Sparsity Enhancement

We define the Capon-like weight as

(20) $$c_{i,j}={\left[C\right]}_{i,j}=b_{i}^{H}{\hat{R}}^{-1}b_{j},$$

and the MUSIC-like weight as

(21) $$d_{i,j}={\left[D\right]}_{i,j}=b_{i}^{H}{\hat{U}}_{N}{\hat{U}}_{N}^{H}b_{j},$$

where *i* and j∈{1,⋯,P}, respectively.

The combination of these two weights is defined as

(22) $$g_{i,j}={\left[G\right]}_{i,j}=\mathrm{Real}\left(c_{i,j}d_{i,j}^{*}\right).$$

Based on these definitions, we can conclude that the quadratic minimization in Equation (17) is a weighted $\ell_{2}$ minimization, whose weights come from the Hadamard product of the Capon-like and the MUSIC-like weighting matrices.

As can been deduced from Equation (20) and (21), if and only if i=j, $c_{i,j}$ and $d_{i,j}$ become the regular Capon weight $c_{i}$ and the regular MUSIC weight $d_{i}$, respectively, where

(23) $$c_{i}=b_{i}^{H}{\hat{R}}^{-1}b_{i},$$

and

(24) $$d_{i}=b_{i}^{H}{\hat{U}}_{N}{\hat{U}}_{N}^{H}b_{i}.$$

The Capon and MUSIC spectral estimations can be directly obtained from $1/\mathrm{Real}(c_{i})$ [3] and $1/\mathrm{Real}(d_{i})$ [6], respectively. Incidentally, similar to Capon and MUSIC [45], the proposed SANSF cannot deal with coherent sources, which is a disadvantage in comparison to the original OWNSF.

Next, we will show that the proposed weighting scheme is consistent with the principle of the weighted $\ell_{p}$ minimization method [7,46,47,48,49]. Namely, the entries whose indices are more likely to be outside of the support are assigned larger weights so that they are suppressed to near zero, while the entries whose indices are more likely to be inside of the support are assigned smaller weights so that they are preferentially recovered. Without loss of generality, the index set {1,⋯,P} is divided into Ω and $\Omega^{c}$, i.e., $\Omega \cup \Omega^{c}=\left\{1,\cdots ,P\right\}$ and $\Omega \cap \Omega^{c}=\emptyset$. Rewriting the objective function of SANSF in Equation (17), we have

(25) $$\begin{array}{ccc} \gamma^{T}G\gamma & = & \sum_{i=1}^{P}\sum_{j=1}^{P}g_{i,j}\gamma_{i}\gamma_{j}\\ & = & \sum_{i\in \Omega }\sum_{j\in \Omega }g_{i,j}\gamma_{i}\gamma_{j}+\sum_{i\in \Omega^{c}}\sum_{j\in \Omega }g_{i,j}\gamma_{i}\gamma_{j}+\sum_{i\in \Omega }\sum_{j\in \Omega^{c}}g_{i,j}\gamma_{i}\gamma_{j}+\sum_{i\in \Omega^{c}}\sum_{j\in \Omega^{c}}g_{i,j}\gamma_{i}\gamma_{j}. \end{array}$$

A proof in Appendix A shows that $\left\{|g_{i,j}|,i\in \Omega \;\mathrm{or}\;j\in \Omega \right\}$ are smaller than $\left\{|g_{i,j}|,i\in \Omega^{c}\;\mathrm{and}\;j\in \Omega^{c}\right\}$. It can be observed from Equation (25) that the set of smaller weights $\left\{g_{i,j},i\in \Omega \;\mathrm{or}\;j\in \Omega \right\}$ and the signal-related items $\left\{\gamma_{i}\gamma_{j},i\in \Omega \;\mathrm{or}\;j\in \Omega \right\}$ are linked. Assigning the set of smaller weights $\left\{g_{i,j},i\in \Omega \;\mathrm{or}\;j\in \Omega \right\}$ to each signal-related item raises the priority of the spectrum peak $\left\{\gamma_{i},i\in \Omega \right\}$ or $\left\{\gamma_{j},j\in \Omega \right\}$ in the recovery process. The positions that more likely correspond to signal-unrelated entries, i.e., $\left\{\gamma_{i},i\in \Omega^{c}\right\}$ and $\left\{\gamma_{j},j\in \Omega^{c}\right\}$, are forced to near zero by weighting with larger values $\left\{g_{i,j},i\in \Omega^{c}\;\mathrm{and}\;j\in \Omega^{c}\right\}$. Therefore, G links smaller/larger weights with the signal-related/signal-unrelated items.

The constraints of SANSF contain two part, i.e., ${∥\gamma ∥}_{1}=\mathrm{Tr}\left\{\Lambda_{S}-\sigma^{2}I\right\}/M$ and γ≥0, which can also promote the sparsity of the solution. The sparsity enforcing property of the $\ell_{1}$ norm constraint is natural because it is the closest convex relaxation of the exact sparse $\ell_{0}$ norm constraint [50]. Moreover, theoretical results in [51,52] show that the non-negative constraint is very helpful for sparsity-promoting. Therefore, combining the weighted quadratic objective function and the $\ell_{1}$ norm and the non-negative constraints, SANSF has a remarkable sparsity-promoting property.

## 4. Numerical and Experimental Results

In this section, some numerical results based on simulated data and real measured ultrasonic data are presented to demonstrate the performance of the proposed SANSF algorithm. The results of several DOA estimation methods, including MUSIC [6], WGMF [11], LIKES [30], and MODE [5], are also provided for comparison purposes. Here, the number of the inner iterations of LIKES is 150, and the number of iterations of MODE is 30 and its initial estimates comes from MUSIC. SANSF is implemented in Matlab function *quadprog* with default options except *OptimalityTolerance* is set to $1e^{-10}$. In all simulations, we consider a linear array with M=10 sensors located at [0.5,1.0,3.0,3.5,6.0,6.5,7.0,7.5,8.0,10.0] in half-wavelength units, unless specified otherwise.

### 4.1. Root-Mean-Squared-Errors (RMSEs)

**Figure 1:**
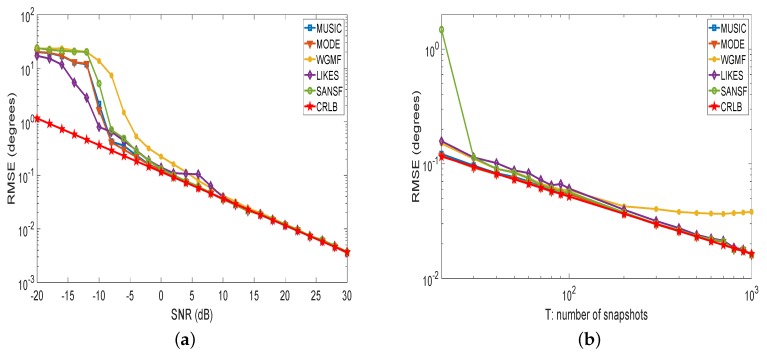
Root-mean-squared-error (RMSE) performance of direction-of-arrival (DOA) estimates comparison. (**a**) RMSE versus SNR with the number of snapshots is 200; (**b**) RMSE versus the number of snapshots with SNR is 10 dB. The number of sensors is 10 and each point is the average of 500 Monte-Carlo trials.

In the first experiment, the root-mean-squared-errors (RMSEs) of the DOA estimates of these methods are compared. Three uncorrelated sources with the same amplitude are impinging on the array from {−34.2°,1.5°,35.8°}. For comparison of the RMSEs of the five algorithms and CRLB, the grid refinement method is used to achieve better precision [8,10,53]. The initial searching space of [−90°,90°] is uniformly sampled by 1126 grid points with the grid spacing of 0.16°. Following the coarse search, three successive iterations are used to further refine the grid spacing, where the local interval around the *k*-th peak of the spectrum, i.e., $\left[{\hat{\theta }}_{k}^{\left(\tau -1\right)}-\frac{8{}^{\circ}}{4^{\tau -1}},{\hat{\theta }}_{k}^{\left(\tau -1\right)}+\frac{8{}^{\circ}}{4^{\tau -1}}\right]$, is uniformly sampled by 401 grid points, ${\hat{\theta }}_{k}^{\left(\tau -1\right)}$ is the estimate of the *k*-th source at step τ−1 (e.g., ${\hat{\theta }}_{k}^{\left(0\right)}$ is the coarse estimate of the *k*-th source), and τ=1,2,3. As shown in Figure 1a, SANSF can reach CRLB with lower SNR than WGMF and LIKES. In addition, as can be seen from Figure 1b, with the increasing number of snapshots, MUSIC, MODE, LIKES, and SANSF can reach the CRLB, but a large number of snapshots is required for LIKES. From Figure 1b it is also noted that the proposed SANSF algorithm is slightly worse than WGMF and LIKES when *T* is not sufficiently large. The reason is that the Capon-like and the MUSIC-like matrices fail to provide reliable weighting values for small values of *T* [54,55]. Especially, the proposed SANSF algorithm does not work for T<M. Furthermore, in contrast to MUSIC, LIKES, and SANSF that exploit the grid refinement method, WGMF is not able to reach the CRLB when the number of snapshots is large enough with the same grid refinement scheme. This fact shows that WGMF requires more sophisticated operations.

### 4.2. Resolution

#### 4.2.1. Probability of Resolution

**Figure 2:**
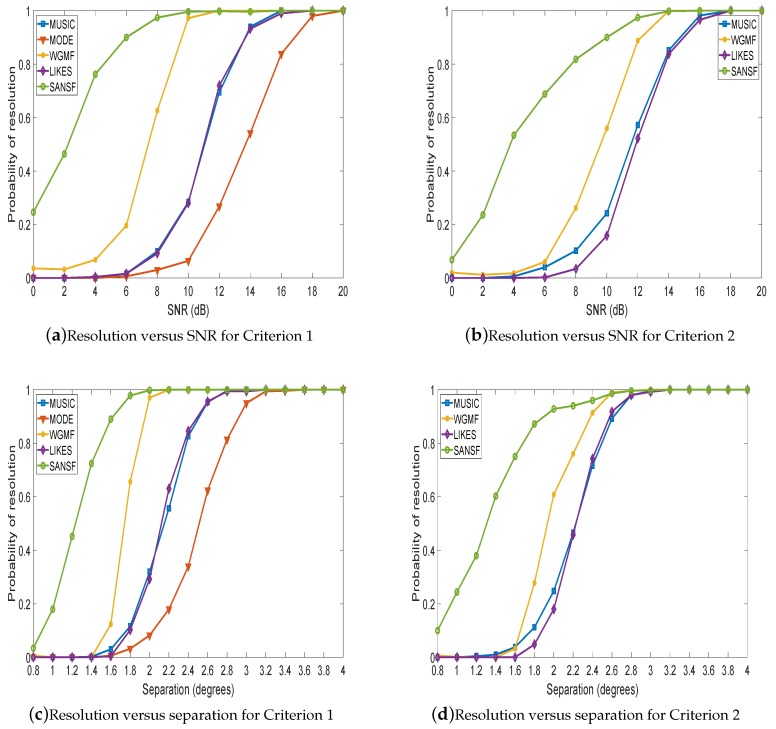
The probability of resolution versus SNR/separation. The separation is 2° for (**a**,**b**); the SNR is 10 dB for (**c**,**d**). The number of snapshots is 200, and each point is the average of 500 Monte-Carlo trials.

In the second experiment, the spatial resolution of SANSF is investigated. The azimuth interval [−90°,90°] is uniformly sampled with 1801 grids. Two closely spaced sources are located at ±Δ/2, where Δ denotes the source separation. Two widely used criteria of resolution are employed. The definition of Criterion 1 [56,57] is that two sources are successfully resolved provided that the estimate ${\hat{\theta }}_{k}$ is located in the neighborhood of the true position $U(\theta_{k};\eta )$. Here, the radius of the neighborhood η is set as η=0.49Δ. As for Criterion 2 [58], a solution would be declared as the successful resolution if and only if $\left[\hat{\gamma }\left(\theta_{1}-r:\theta_{1}+r\right)+\hat{\gamma }\left(\theta_{2}-r:\theta_{2}+r\right)\right]/\left(4r+2\right)-\hat{\gamma }\left(0\right)>0$, where *r* denotes the searching radius of estimated peaks (in this paper, r=3) (Here, the definition of Criterion 2 is slightly modified by considering the searching radius of the estimated peaks. If r=0, it is exactly the same as the original definition in [58].). The estimate of *K* comes from the MDL criterion [39]. As can be seen from Figure 2 (In Figure 2b,d, the results of MODE are absent because MODE does not yield the spatial spectrum that is necessary for Criterion 2. MODE will not be present in Figures 4 and 5 for the same reason.), SANSF has higher probability of successful resolution than the other four methods. The reason is that the sparsity of the solution of SANSF is enhanced by embedding the weighting matrix G and forcing the $\ell_{1}$ norm and the non-negative constraints. It is also noted that MODE cannot yield satisfactory resolution performance in these experimental results because the initialization value from MUSIC may be inaccurate for closely spaced sources.

#### 4.2.2. Histogram for 1D Closely Spaced Sources

**Figure 3:**
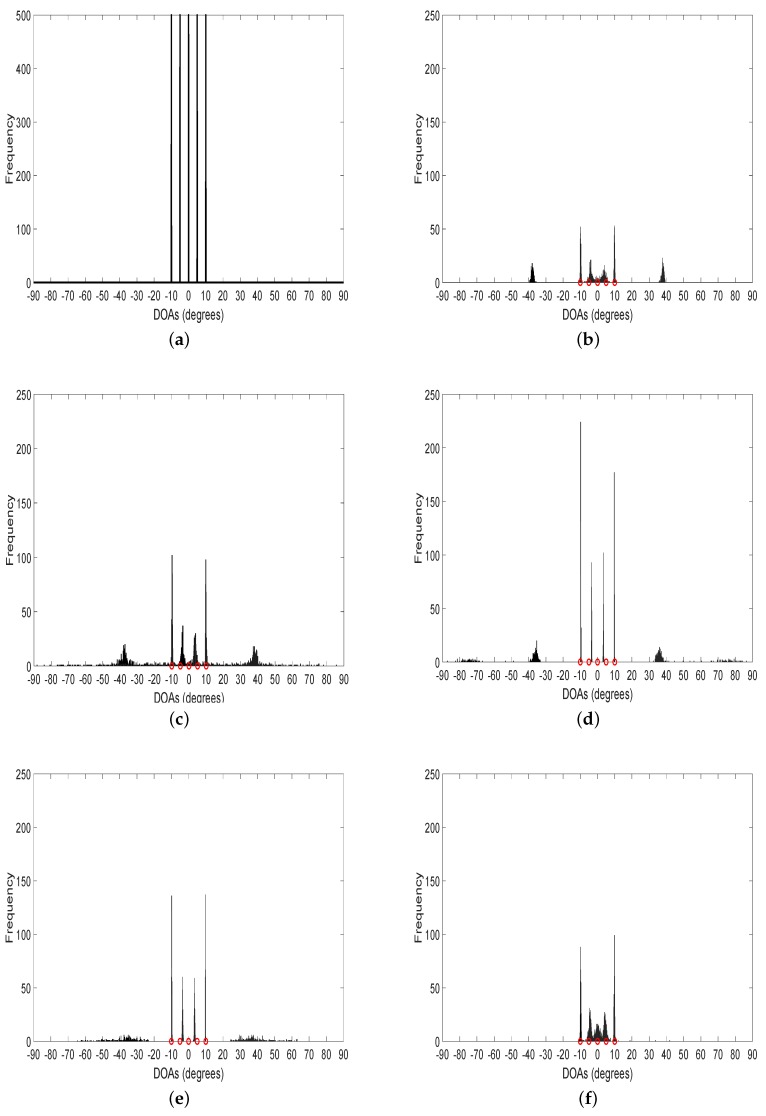
The histogram over 500 Monte-Carlo trials. The number of snapshots is 500, SNR is 20dB. The five red circles denote the true positions of the sources. (**a**): True; (**b**): MUSIC; (**c**): MODE; (**d**): WGMF; (**e**): LIKES; (**f**): SANSF.

In the third experiment, we compare the 1D resolution in the signal-dense situation. The histogram of DOA estimates over 500 Monte-Carlo trials are plotted in Figure 3, where the azimuth interval [−90°,90°] is uniformly sampled with 1801 grids and five uncorrelated and closely spaced sources with the same amplitude exist on the grid points [801,851,901,951,1001]. As demonstrated in Figure 3, histograms of MUSIC, MODE, WGMF, and LIKES show only four peaks around the true positions of the sources and a number of spurious peaks. This indicates that they fail to deal with the signal-dense situation. It is noteworthy that the proposed SANSF algorithm clearly shows five peaks at the true positions of the sources.

#### 4.2.3. Spatial Spectrum for 2D Closely Spaced Sources

**Figure 4:**
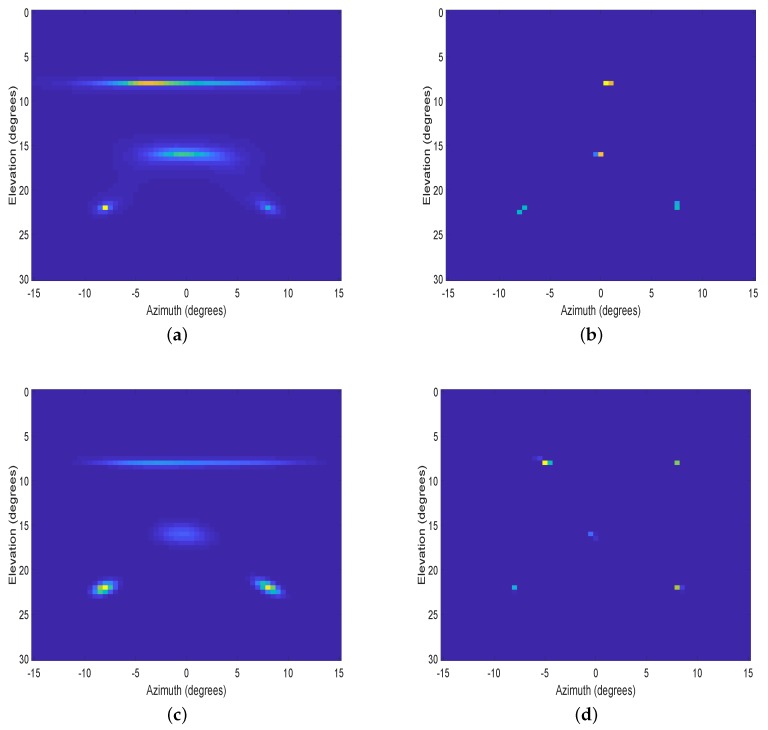
Top view of the normalized 2D spatial spectrum with an L-shaped array. DOAs: (−6°,8°), (6°,8°),(0°,16°), (−8°,22°), and (8°,22°). (**a**): MUSIC; (**b**): WGMF; (**c**): LIKES; (**d**): SANSF.

**Figure 5:**
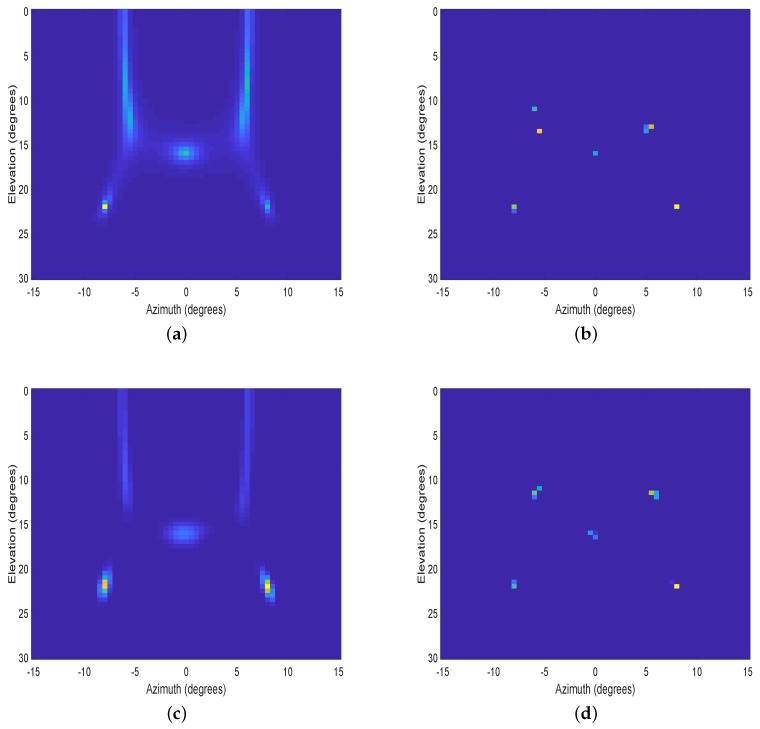
Top view of the normalized 2D spatial spectrum with an uniform circular array. DOAs: (−6°,8°), (6°,8°),(0°,16°), (−8°,22°), and (8°,22°). (**a**): MUSIC; (**b**): WGMF; (**c**): LIKES; (**d**): SANSF.

In the fourth experiment, two top views of the 2D spatial spectrum are used to further demonstrate the resolution performance of SANSF in the signal-dense situation. Five uncorrelated sources with amplitudes (15,15,15,15,15) from (−6°,8°), (6°,8°),(0°,16°), (−8°,22°), and (8°,22°) are considered, where the number of snapshots is 800. The azimuth interval [−15°,15°] and the elevation interval [0°,30°] are uniformly sampled with 61 grids, respectively. In Figure 4, we use an L-shaped array consisting of six horizontal array elements and five vertical array elements with half-wavelength spacing. As shown in Figure 4, the proposed SANSF algorithm clearly shows five peaks with no visible artifacts, while other algorithms are failing to resolve all of them. In Figure 5, a uniform circular array (UCA) consisting of 16 elements with half-wavelength spacing is employed to obtain the spatial spectrum. Again, the resolution performance of the proposed SANSF algorithm outperforms other algorithms. Especially, Figure 4a and Figure 5a, and Figure 4c and Figure 5c, show that MUSIC and LIKES suffer from low-resolution and high-sidelobes. Although WGMF has higher resolution than MUSIC and LIKES, as can be seen from Figure 4b and Figure 5b it is apt to underestimate the sources for L-shaped array and may suffer from undesired artifacts for UCA in the signal-dense situation.

### 4.3. Robustness to the Assumption of Uncorrelated Sources

**Figure 6:**
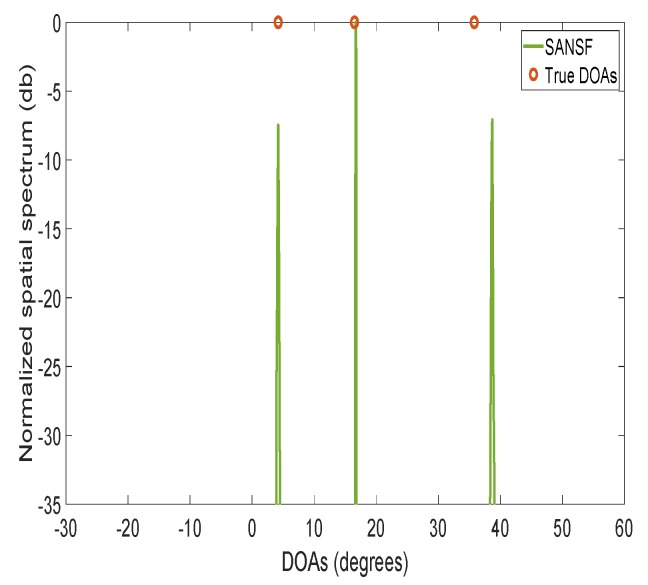
Normalized spatial spectrum of three correlated sources. M=10, SNR is 20 dB, T=200, DOAs: {4.2°,16.5°,35.8°}, and each point is the average of 100 Monte-Carlo trials.

In the fifth experiment, we consider the correlated sources scenario, where three correlated sources have the same amplitude with the correlation coefficient matrix [1,0.8,0.9;0.8,1,0.8;0.9,0.8,1]. In Figure 6, the averaged spatial spectrum over 100 Monte-Carlo trials is plotted. It is obvious that the proposed SANSF algorithm can deal with the correlated sources and is robustness to the assumption of uncorrelated sources. A detailed explanation for the robustness can be found in the literature [45,59].

### 4.4. Computational Complexity

**Figure 7:**
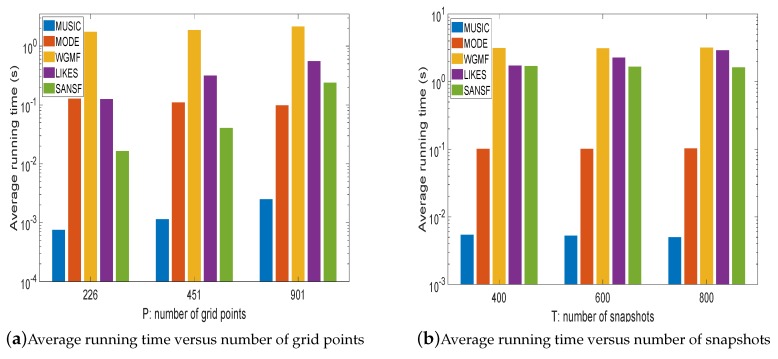
Bar plot of the average running time. The number of sensors is 10, SNR is 10 dB, the number of sources is 3, and each bar is the average of 500 Monte-Carlo trials, the number of snapshots is set to 200 for (**a**), the number of grid points is set to 1801 for (**b**).

In the sixth experiment, the average running time yielded by the above methods (i.e., MUSIC, MODE, WGMF, LIKES, and SANSF) are compared, where all the experiments are implemented with Matlab 2018b on a PC with the Intel CORE i5-7200U CPU @2.50GHz. Figure 7 shows that, compared to WGMF and LIKES, the proposed SANSF has a lower computational burden, especially for the smaller number of grid points. It is also noted that, with increasing number of snapshots, the computational complexities of WGMF and SANSF only slightly increase. Although MUSIC has the lowest computational cost among these algorithms, its resolution is not competitive as shown in Figure 2, Figure 3, Figure 4 and Figure 5.

### 4.5. DOA Tracking Results with Real Measured Ultrasonic Data

The real measured ultrasonic data are from the University of Wyoming Source Tracking Array Testbed (UW STAT) [60]. Here, the data set Number 2, that has been used for evaluating DOA performance in [61,62], is employed. One stationary source and one moving source were present in the scene, the number of sensors was 6, the number of snapshots was 1533, the carrier frequency was 40 KHz, and the signal bandwidth was 200 Hz. The sensor array was an ULA with the interelement spacing 2.1 times of the wavelength. The sliding window technique with the size of 150 snapshots and the forward–backward smoothing are used to obtain the source trajectories.

**Figure 8:**
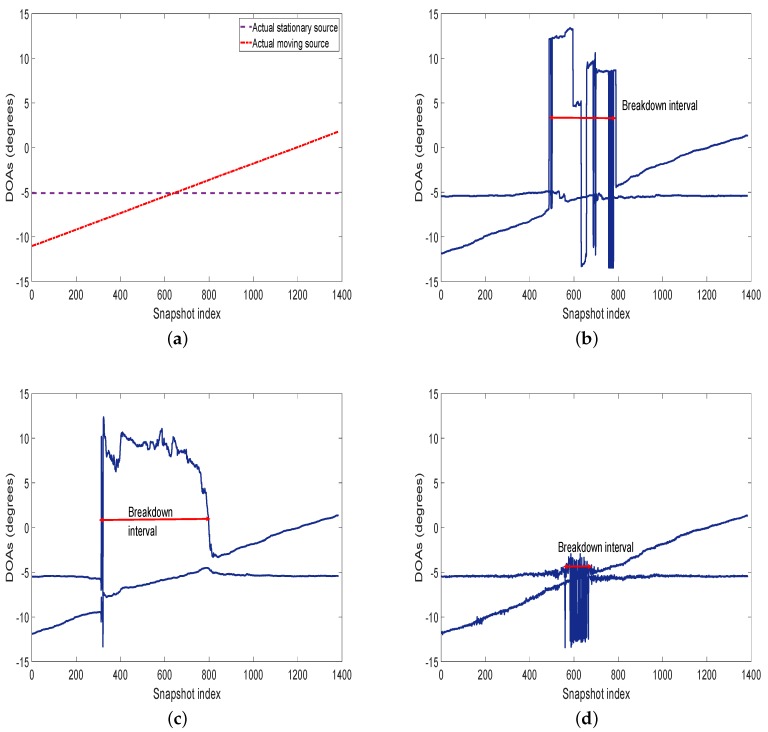

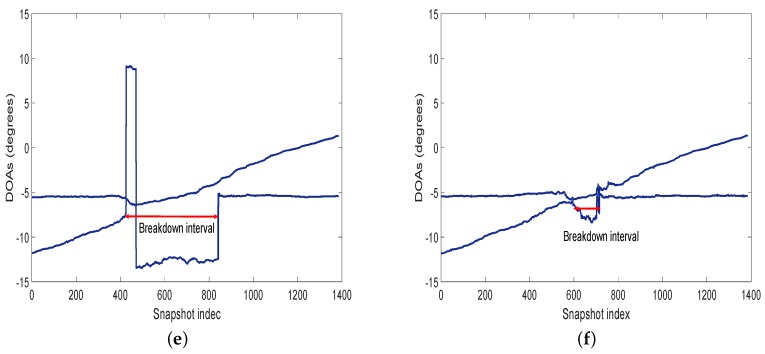
Estimated trajectories versus the snapshot index for the real ultrasonic data. The red double arrow solid line denotes the breakdown interval. (**a**): Actual trajectories; (**b**): MUSIC; (**c**): MODE; (**d**): WGMF; (**e**): LIKES; (**f**): SANSF.

From Figure 8, it is obvious that not all methods are capable of dealing with the intricate scene that the sources are very closely spaced. However, one can observe that SANSF has better threshold performance than the other three methods. The algorithm performance would “break down” when these two sources are too closely spaced [61]. The performance degradation interval due to the sources becoming closely spaced is called a breakdown interval. The breakdown interval shows the ability of the method to resolve the closely spaced sources. The smaller breakdown interval is, the higher the resolution. Table 1 provides the breakdown interval of these methods. Compared with the other methods, SANSF has the narrowest breakdown interval which is about 114 snapshots. This further demonstrates that SANSF can yield superior resolution performance in practice.

### 4.6. DOA Eestimation Results with Real Radar Data

**Figure 9:**
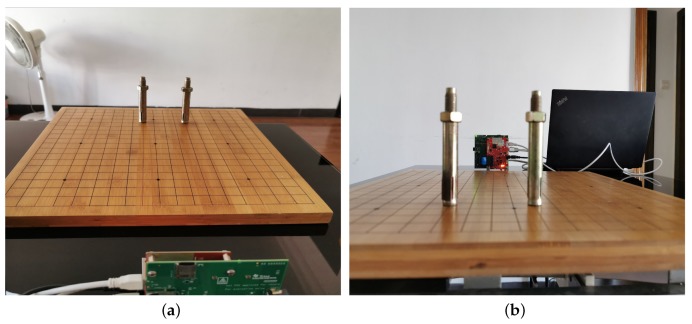
Targets scene consisting of two closely spaced screws. (**a**): Front view; (**b**): Rear view.

The cross-range imaging of radar can be regarded as the DOA estimation problem in the azimuth dimension [63]. Therefore, we can evaluate the DOA estimation resolution by the cross-range resolution. The real radar data were collected by the TI IWR1642BOOST evaluation board that is a frequency modulated continuous wave (FMCW) sensor. The sensor configuration is shown in Table 2, in which four receivers and one transmitter form a four-element ULA. As seen from Figure 9, two screws with a diameter of about 1.5 cm and a height of 11 cm were used as observation targets. The distance from the radar to the midpoint between the two targets is about 50 cm. Center-to-center cross-range spacings between these two screw targets are about 12.5 cm, 15.0 cm, 17.5 cm, and 20 cm, respectively. As a reference, the cross-range Rayleigh resolution limit [63] for our scenario is approximately 12.5 cm.

**Figure 10:**
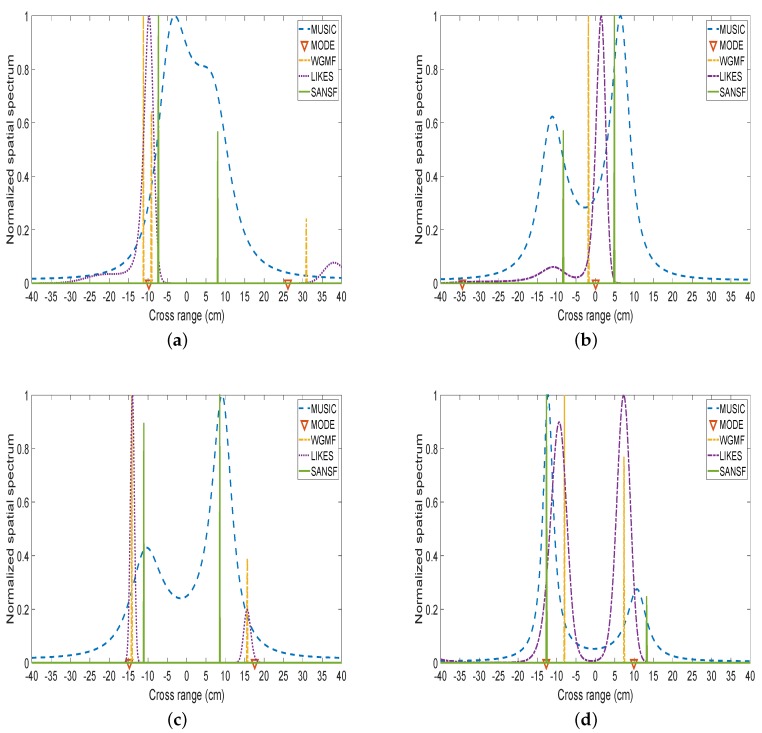
Azimuth resolution versus cross-range spacings between two screw targets for real radar data. The cross-range spacing between these two screws are 12.5 cm for (**a**); 15.0 cm for (**b**) 17.5 cm for (**c**); 20.0 cm for (**d**).

We obtained the cross-range (i.e., azimuth DOAs) of two screws after performing two FFT that were used to estimate the range and velocity information of screws. As can be seen from Figure 10, the proposed SANSF algorithm can deal with the real radar data with higher azimuth resolution compared to other algorithms. However, MODE, WGMF, and LIKES failed to distinguish two closely spaced targets when their range spacings are comparable to the Rayleigh resolution limit. MUSIC distinguishes two closely spaced targets but it has higher sidelobes. Obviously, all methods can clearly distinguish two screws when their cross-range spacing are large enough.

## 5. Conclusions

In this paper, a sparsity-aware SANSF algorithm has been introduced for DOA estimation. SANSF is derived from the OWNSF criterion and involves a convex quadratic minimization problem subject to the linear constraints. This allows us to solve SANSF by using LCQP solvers at an acceptable computational cost. A Hadamard product of the MUSIC-like and the Capon-like matrices is embedded into the objective function, which embodies the weighted $\ell_{2}$ minimization methodology. The weighting and the $\ell_{1}$ norm and the non-negative constraints can together enhance the sparsity of the solution. Simulations and experimental results on measured ultrasonic and radar data have demonstrated that the proposed SANSF algorithm has superior resolution performance over typical sparsity-driven methods for noncoherent sources.

## Figures and Tables

**Table 1:** Breakdown interval.

Algorithms	MUSIC	MODE-ULA	WGMF	LIKES	SANSF
Start	488	314	560	426	601
End	789	809	680	847	715

**Table 2:** Radar configuration paramerers.

Parameters	Tx Channel	Rx Channel	Start Frequency	Frequency Slope	ADC Samples	Sample Rate
Value	Tx1	Rx1,2,3,4	77 GHz	66.626 MHz/μs	256	5000 ksps

## Appendix A

In this appendix, we prove that $g_{i,j}\;\mathrm{for}\;i\in \Omega \;\mathrm{or}\;j\in \Omega$ is smaller than $g_{i,j}\;\mathrm{for}\;i\in \Omega^{c}\;\mathrm{and}\;j\in \Omega^{c}$. Without loss of generality, we assume that $i\in \Omega ,\;j\in \Omega^{c},\;\mathrm{and}\;l\in \Omega^{c}$.

First, we discuss the properties of $c_{i,j}$ and $c_{l,j}$. According to Equation (20), we have

(A1) $$\begin{array}{ccc} |c_{i,j}| & = & |b_{i}^{H}\hat{U}{\hat{\Lambda }}^{-1}{\hat{U}}^{H}b_{j}|\\ & = & |b_{i}^{H}{\hat{U}}_{S}{\hat{\Lambda }}_{S}^{-1}{\hat{U}}_{S}^{H}b_{j}+b_{i}^{H}{\hat{U}}_{N}{\hat{\Lambda }}_{N}^{-1}{\hat{U}}_{N}^{H}b_{j}|\\ & \approx & |b_{i}^{H}{\hat{U}}_{S}{\hat{\Lambda }}_{S}^{-1}{\hat{U}}_{S}^{H}b_{j}|, \end{array}$$

and

(A2) $$\begin{array}{ccc} |c_{l,j}| & = & |b_{l}^{H}\hat{U}{\hat{\Lambda }}^{-1}{\hat{U}}^{H}b_{j}|\\ & = & |b_{l}^{H}{\hat{U}}_{S}{\hat{\Lambda }}_{S}^{-1}{\hat{U}}_{S}^{H}b_{j}+b_{l}^{H}{\hat{U}}_{N}{\hat{\Lambda }}_{N}^{-1}{\hat{U}}_{N}^{H}b_{j}|, \end{array}$$

where the approximate equality in Equation (A1) can be obtained by the orthogonality between the signal subspace and the noise subspace. When SNR is sufficiently large, $|c_{i,j}|\approx 0$ and $|c_{l,j}\left|\approx \right|b_{l}^{H}{\hat{U}}_{N}{\hat{U}}_{N}^{H}b_{j}|/{\hat{\sigma }}^{2}\gg 0$.

Next, we discuss the properties of $d_{i,j}$ and $d_{l,j}$. Because of the orthogonality between the signal subspace and noise subspace, we have

(A3) $$\begin{array}{c} |d_{i,j}\left|=\right|b_{i}^{H}{\hat{U}}_{N}{\hat{U}}_{N}^{H}b_{j}|\approx 0, \end{array}$$

and

(A4) $$\begin{array}{c} |d_{l,j}\left|=\right|b_{l}^{H}{\hat{U}}_{N}{\hat{U}}_{N}^{H}b_{j}|>0. \end{array}$$

Substituting Equations (A1)–(A4) into Equation (22), we have $|g_{i,j}|\approx 0$ and $|g_{l,j}|>0$. Therefore, $\left\{|g_{i,j}|,i\in \Omega \;\mathrm{or}\;j\in \Omega \right\}$ is smaller than $\left\{|g_{i,j}|,i\in \Omega^{c}\;\mathrm{and}\;j\in \Omega^{c}\right\}$. □
